# Cervical repetitive peripheral magnetic stimulation relieves idiopathic persistent hiccups: A preliminary study of case report

**DOI:** 10.1097/MD.0000000000031324

**Published:** 2022-10-28

**Authors:** Weisen Cai, Guangqing Xu, Zongguang Tian, Feng Xiong, Jiajing Yang, Tong Wang

**Affiliations:** a Department of Rehabilitation Medicine, The First Affiliated Hospital of Nanjing Medical University, Nanjing, China; b Department of Rehabilitation Medicine, Wuxi Huishan District Rehabilitation Hospital, Wuxi, China.

**Keywords:** hiccups, repetitive peripheral magnetic stimulation (rPMS)

## Abstract

We postulated that repetitive peripheral magnetic stimulation (rPMS) might treat idiopathic persistent hiccups. This study aimed to determine the clinical effect of rPMS on hiccup alleviation.

**Methods:**

Seven patients with idiopathic persistent hiccups experienced the cervical rPMS session (1 Hz, 656 stimuli) in this prospective clinical series from November 2018 to May 2021. The rPMS session was applied once daily until the hiccups were utterly relieved. During the treatment, the round coil was transversally positioned over the upper nape area, and the center of the coil was placed at the level of the C4 vertebrae. The subjective assessment scale (SAS) scores and the hiccup frequency were assessed before and after rPMS treatment.

**Results:**

A total of 7 patients were enrolled. All were male post-stroke patients ([mean ± SD] age, 58.5 ± 9.85 years) with dysphasia, 3 patients (3/7) were fed with a nasogastric tube, and 4 patients (4/7) were with dysarthria. The mean duration of hiccups was 4.14 ± 3.63 days (range 2–12 days). The rPMS therapy eliminated hiccups in all 7 patients. The mean sessions which stopped hiccupping were 3.43 ± 2.57 (range 1–9). The mean value of the SAS scores before rPMS therapy was 7 ± 1 (range 6–8), and it was decreased to zero after the therapy (0). No recurrence of hiccups was observed within 2 weeks of the last rPMS session. rPMS therapies were not associated with severe adverse effects.

**Conclusion:**

The cervical rPMS therapy is beneficial in treating idiopathic persistent hiccups, particularly in post-stroke patients.

## 1. Introduction

Hiccups are caused by a series of involuntary spasmodic contractions of the diaphragm and intercostal muscles, followed by a sudden closure of the glottis, which results in a peculiar “hic” sound.^[1,2]^ It is widely believed that hiccups are caused by a “reflex arc,” which includes an afferent limb, a central connection, an efferent limb, and effectors.^[3,4]^ Typically, hiccups occur in cycles of 4 to 60 per minute and could be considered a form of diaphragmatic rhythmic myoclonus.^[5]^ Golomb described the hiccup as a respiratory arrhythmia rather than reflexes.^[6]^ Clinically, the classification of hiccups is based on their duration.^[7]^ Transient hiccups last less than 48 hours, persistent hiccups last more than 48 hours, and intractable hiccups last more than 1 month.^[4]^ Pathological factors may bring on long-lasting and challenging-to-cure hiccups. Idiopathic hiccups refer to hiccups for which the cause cannot be identified.^[8]^ Persistent or intractable hiccups can cause exhaustion and anxiety, even make the underlying condition worse.^[9]^ Thus, it should be addressed.

Multiple different medications and non-pharmacological interventions (such as physical maneuvers and the phrenic nerve blockade) have been used to stop persistent or intractable hiccups. However, the “perfect method” (a specific treatment for hiccups) has not yet been discovered, and structured guidelines have not yet been established.^[9]^

Repetitive peripheral magnetic stimulation (rPMS) has been effectively used to treat low back pain by regulating the function of the peripheral nerve and muscles.^[10–12]^ Cervical magnetic stimulation, which stimulates the phrenic nerves, has been shown to cause bilateral diaphragm contractions.^[13]^ Therefore, we hypothesized that magnetic stimulation might prevent hiccups when rPMS is applied to the neural structures that comprise the hiccup reflex arc. Furthermore, no reports of adverse reactions to rPMS have been found.^[12]^ Therefore, rPMS can be considered a safe and feasible method to treat hiccups. This study aimed to determine the clinical effect of rPMS on hiccup alleviation.

## 2. Materials and Methods

### 2.1. Subject recruitment

Seven hospitalized patients with idiopathic persistent hiccups were enrolled in the study between November 2018 and May 2021. All patients underwent a systemic examination to rule out the contraindications to rPMS. Conditions that may cause hiccups have not been identified. The preconditions for acceptance in this study were that the hiccups were diagnosed as idiopathic hiccups lasting at least 48 hours. Before rPMS therapy, physical maneuvers or medication for hiccups were allowed. The periods between previous empiric therapy (baclofen or metoclopramide) and rPMS therapy were at least 12 hours to exclude the influence of these medications. Prior to and following rPMS therapy, the frequency and severity of hiccups were measured using a subjective assessment scale (SAS), which ranged from 0 (no hiccupping) to 10 (unbearable hiccupping).^[14]^

This study was approved by the Institutional Ethics Committee of the hospital (HK-LLWYH-2018013), which adheres to the tenets of the Declaration of Helsinki. Patients provided informed consent to participate in the study. Written informed consent was received from the patients for publication of the data and any accompanying images.

### 2.2. Rpms procedures

**Figure 1.**
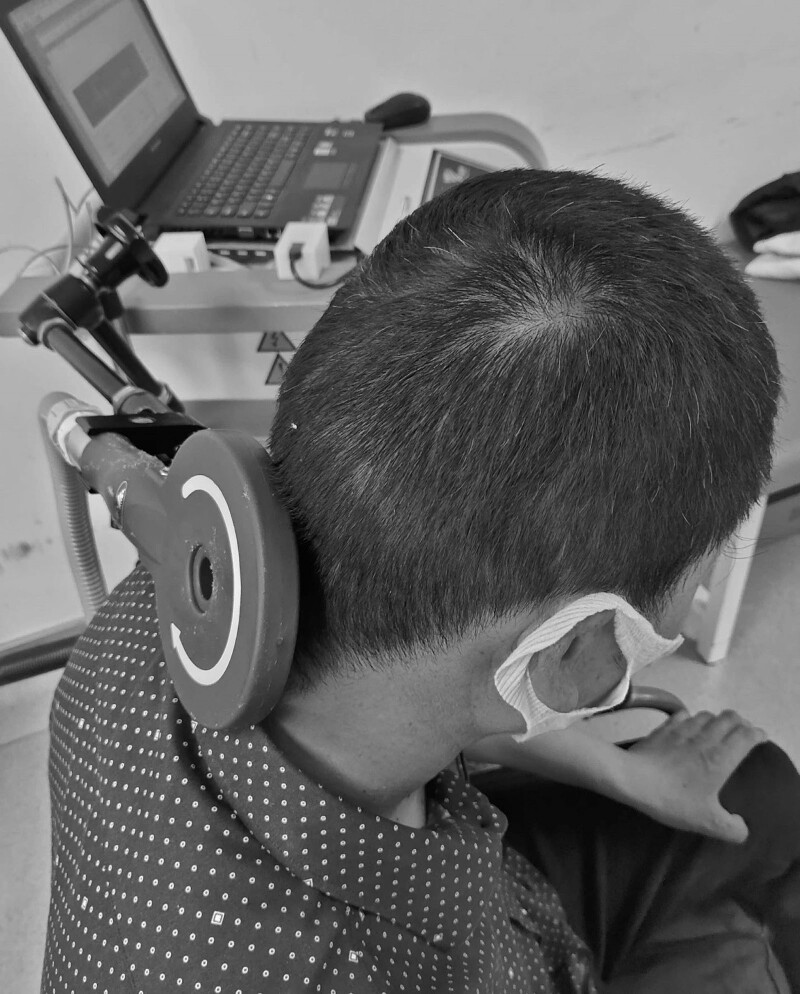
Treatment of details.

In this study, a magnetic stimulator with a round coil, model CCY-1 (Yiruide Medical Equipment Company, China), was employed. The parameter of rPMS sessions followed the security guidelines and protocols. The treatment was carried out in a comfortable and quiet room. The round coil was transversally positioned over the upper nape with the help of a metal arm. The center of the round coil was positioned over the C4 vertebra, for it is where the hiccup reflex arc center located, and the nerve roots of phrenic nerves come from. In the seated position, the patient’s head should be bent forward as much as feasible to eliminate the consequences of the cervical lordosis (Fig. 1).

Prior to treatment, the resting motor thresholds (RMT) of patients were determined in order to evaluate the excitability of each patient’s nervous system, as in repetitive transcranial magnetic stimulation therapy. The RMT was measured by placing the round coil over the primary motor cortex (M1) of the unaffected cerebral hemisphere. The RMT was defined as the 90% of the lowest stimulation intensity capable of eliciting the slightest observable movement of the accompanying hand or fingers. Parameters of the 15-minute stimulation protocol consisted of 82 trains of 8 pulses at a frequency of 1 Hz, followed by a 3-second inter-train interval (total of 656 pulses) at a tolerable intensity (80%–120% of the RMT). Patients received an rPMS session once a day. The rPMS therapy was planned to continue until the hiccupping stop. If hiccups reoccur within the following 2 weeks, rPMS therapy will be applied again. The SAS was assessed, and the adverse effects were also observed. Total hiccup relief was defined as having no hiccups within 2 weeks and receiving a score of 0 for both SAS and hiccup frequency.

## 3. Results

The history of hiccups and treatment details are provided in Table 1. All patients were male post-stroke, and 2 patients (2/7) had a minimally conscious state. All the patients had dysphasia; 3 (3/7) required nasogastric feedings, and 4 (4/7) had dysarthria. The mean age of the patients was 58.5 ± 9.85 years (range 46–72 years). The mean length of stroke history in the 7 study patients was 6.14 ± 4.34 months (range 2–12 months). The mean length of hiccup history was 4.14 ± 3.63 days (range 2–12 days), and all 7 patients had hiccups for 24 hours. The pretreatment hiccup rate was given in the form of a range. The low mean was 11.43 ± 3.78 (range 5–15) hiccups per minute, and the upper mean was 22.14 ± 6.36 (range 10–30) hiccups per minute. The SAS score of hiccups was assessed before and after therapy. There were only 5 SAS data available since 2 patients had minimally conscious state. The mean value of SAS was 7 ± 1 (range 6–8).

**Table 1:** The patients’ information and treatment details.

Patient no. (Gender/age)	Medical history	Hiccup duration*	Previous medication for hiccup	Hiccup frequency (SAS)**		Number of rPMS sessions
				Before therapy	After therapy	
1. (M/46 yr)	Hemorrhage (6 mo), hemiplegia (R), dysphagia (nasogastric tube), dysarthria	12 d, 24 h/d	Baclofen 10 mg 3 times	10–30 (7)	0 (0)	9
2. (M/59 yr)	Hemorrhage (6 mo), MCS, dysphagia (nasogastric tube)	5 d, 24 h/d	-	10–25 (-)	0 (-)	3
3. (M/46 yr)	TBI (12 mo), MCS, dysphagia (nasogastric tube)	3 d, 24 h/d	Baclofen 10 mg 3 times	15–20 (-)	0 (-)	3
4. (M/72 yr)	Ischemic (2 mo), hemiplegia (R), dysphagia	2 d, 24 h/d	Metoclopramide 10mg im, once	10–25 (8)	0 (0)	3
5. (M/65 yr)	Ischemic (3 mo), hemiplegia (L※), dysphagia, dysarthria	2 d, 24 h/d		5–10 (6)	0 (0)	1
6. (M/57 yr)	Hemorrhage (12 mo), hemiplegia (R), dysphagia, dysarthria	2 d, 24 h/d		15–20 (6)	0 (0)	2
7. (M/65 yr)	Hemorrhage (2 mo), hemiplegia (R), dysphagia, dysarthria	3 d, 24 h	Baclofen 10 mg twice, metoclopramide 10 mg im, twice	15–25 (8)	0 (0)	3

d = days, h/d = hours/days, L = left, m = months, M = male, MCS = minimally conscious state, R = right, TBI = traumatic brain injury.

* Hiccup duration is length of hiccup history given as days (d), duration of hiccup episodes given as hours per day (h/d).

** SAS score: 0 = no hiccupping, 10 = unbearable hiccupping; 2 patients’ SAS score could not get for they were MCS.

During the hiccupping days preceding rPMS treatment, all 7 patients sought to apply pressure on the eye, take baclofen orally, or inject metoclopramide intramuscularly, but none of them were successful.

The rPMS therapies were not associated with severe adverse effects. Following rPMS treatment, all 7 patients were completely free of hiccups. The mean SAS scores before and after therapy were (7 ± 1) and (0), respectively. The average number of rPMS sessions required for these patients to stop hiccupping was 3.43 and 2.57 (range 1–9). Each session of rPMS could give at least part of hiccup relief. The No. 5 patient experienced complete hiccup resolution following just 1 session of rPMS therapy. No hiccup recurrence was noticed within 2 weeks after the final rPMS session.

## 4. Discussion

In the present study, we assessed the effectiveness of rPMS in treating hiccups. All hiccups in the survey disappeared, with no severe adverse effects and no recurrence within 2 weeks.

All 7 of the patients with idiopathic persistent hiccups in this study were men who had recently experienced a stroke and had dysphasia; 4 patients (4/7) also had dysarthria, and 3 patients used a nasogastric tube. Thus, the male, post-stroke patients, especially those with dysphasia and dysarthria, may be more susceptible to idiopathic persistent hiccups. It is consistent with the findings of previous researchers who found that idiopathic hiccups are assumed to be caused by the digestive system or the central nervous system.^[4,7]^ Male patients are more likely to suffer from hiccups.^[15]^ The findings of this investigation revealed that the neural tissue dysfunction might be the idiopathic hiccups in post-stroke patients.

The results showed that the rPMS therapy could relieve hiccups immediately and keep no hiccupping in the following 2 weeks. The effects of the rPMS therapy on hiccups are like its impact on low back pain. The evidence indicated that rPMS therapy could relieve hiccups or pain through a short-term or long-term mechanism. The long-term mechanism of hiccup relief is uncertain, despite some rPMS studies on the reduction of low back pain attributing pain reduction to proprioceptive-induced brain reorganization.^[12]^

In this study, the rPMS could penetrate to the deep structures beneath the skin, form electrical fields, depolarize the phrenic nerves,^[12]^ and then trigger trains of diaphragm contraction. We adopted the low frequency rPMS at a frequency of 1 Hz, which was higher than the upper mean value of the hiccup rate (22.14 ± 6.36 per minute, range 10–30). As a result, the rPMS-induced diaphragm contraction may suppress the rhythmic diaphragm contraction associated with hiccups, subsequently ceasing the hiccups. The overdrive suppression phenomenon occurred after hiccup relief, which may be analogous to what happened during cardiac arrhythmia treatment.^[16]^ The overdrive suppression mechanism may be the most plausible explanation for the results of this investigation. Whether the hiccups are controlled by a Glomb-described respiratory rhythm center or an unidentified hiccup rhythm center is uncertain.^[6]^ It is also uncertain what causes the overdrive suppression phenomena, whether it is the frequency or the intensity of the magnetic stimulation. If a mechanism of overdrive suppression inhibition exists, studies employing a higher frequency of rPMS treatment may be more fruitful.

According to the findings, rPMS therapy provided 100% (7/7) alleviation from hiccups with a short course of treatment (mean number of sessions, 3.43 ± 2.57; range 1–9). Therefore, rPMS therapy is an effective way to eliminate hiccups. Hundreds of basic physical manipulations aim to stop hiccups by interrupting or suppressing the reflex arc,^[4]^ such as holding one’s breath, applying pressure on the eyes, massaging the carotid massage, and self-induced vomiting.^[4]^ These noninvasive techniques were always used before the medication or invasion therapies.^[9]^ As for the persistent and intractable hiccups, these physical manipulations are undoubtedly a failure. Medication is the primary therapy for persistent or intractable hiccups. The medicines such as metoclopramide, baclofen, gabapentin, and pregabalin are for hiccups originating in the central nervous system.^[14]^ And the medicines such as cisapride and omeprazole are for hiccups originating in the digestive system.^[14]^ However, a Cochrane systemic review concluded inadequate evidence to support the notion that pharmacological medicines are the most effective treatment for hiccups.^[17]^ Georg applied a combination therapy with cisapride, omeprazole, and baclofen for the idiopathic chronic hiccup, 60% of patients gained substantial relief or even better (disappearance), but the poor compliance and the long course (12 months) of treatment attempts are the limiting factors.^[14]^ Phrenic nerve blockade or radiofrequency ablation was even regarded as a “final resort” to treat intractable hiccups.^[4,18]^ However, since the phrenic nerve is not the only nerve innervating the diaphragm, sometimes this intervention can fail even if both sides of the phrenic nerve are blocked.^[19,20]^ And cases of acute dyspnea respiratory compromise after phrenic nerve blockade have been reported.^[21–23]^ Therefore, the phrenic nerve block cannot be regarded as an ideal technique for hiccup elimination. Moreover, the blockade can be merely operated by a well-experienced anesthetist, these negative factors limit clinical application. Consequently, cervical rPMS therapy for hiccups is more effective and practical than medication therapy and intervention therapy.

Under the circumstances of this study, neither double-blind nor placebo controls were feasible. Therefore, this study was not masked or placebo controlled. Consequently, one of the limitations of our study is that we cannot rule out the coincidence and placebo effect as a possible explanation for eliminating hiccups. In addition, the limited sample size and homogeneous patients may restrict the generalizability of the study’s findings.

## 5. Conclusion

Cervical rPMS therapy appears to be a safe, workable, and effective treatment for idiopathic persistent hiccups, particularly in post-stroke patients. Since the mechanism remains unclear, further research should be conducted.

## Author contributions

**Conceptualization:** Weisen Cai.

**Data curation:** Zongguang Tian, Feng Xiong.

**Formal analysis:** Jiajing Yang.

**Investigation:** Weisen Cai, Guangqing Xu.

**Software:** Zongguang Tian, Feng Xiong, Jiajing Yang.

**Writing – original draft:** Weisen Cai, Guangqing Xu.

**Writing – review & editing:** Tong Wang.

## Correction

Dr. Weisen Cai’s name has been corrected from Weiseni Cai.
